# Nanocomposite Chitosan@graphene Oxide-Based Aerogel Beads for Anionic and Cationic Dye Removal: Synthesis, Characterizations, and Complexed Interfacial Interactions in Batch and Column Studies

**DOI:** 10.1021/acsomega.5c04669

**Published:** 2025-07-08

**Authors:** Shehryar Ahmad, Enrica Luzzi, Konstantinos N. Maroulas, George Z. Kyzas, Martina Salzano de Luna

**Affiliations:** a Department of Chemical, Materials, and Production Engineering (INSTM Consortium − UdR Naples), 9307 University of Naples Federico II, P.le Tecchio 80, Naples 80125, Italy; b Hephaestus Laboratory, School of Chemistry, Faculty of Sciences, 37791 Democritus University of Thrace, Kavala GR-65404, Greece

## Abstract

One of the greatest global threats to the environment and human health is the persistent pollution of water. Adsorption is a recognized remediation method that effectively and efficiently removes pollutants from water. In this work, nanocomposite aerogel beads of chitosan-graphene oxide (CS/GO) and chitosan-reduced graphene oxide (CS/rGO) were synthesized by a simple three-step procedure and evaluated as an adsorbent material for water purification. In particular, two model pollutant molecules, namely, indigo carmine (IC, anionic dye) and methylene blue (MB, cationic dye), were used to study the adsorption behavior of the developed materials by kinetics and isothermal analyses in batch configuration. Adsorption kinetics follows the Elovich model well, while adsorption isotherms are better described by the Freundlich model. CS/GO aerogel beads had greater MB affinity, while CS/rGO exhibited better IC adsorption removal, with maximum adsorption capacities of 254 and 109 mg g^–1^, respectively, according to Langmuir fitting. In view of this result, the adsorption behavior of mixed CS/GO and CS/rGO beads at 1:3, 1:1, and 3:1 weight ratios was also investigated with the aim of achieving broad-spectrum removal capacity, that is, the ability to remove both anionic and cationic species at the same time. In addition, a custom-made fixed-bed configuration at a 15 mL scale was exploited to evaluate the adsorption capacity of CS/GO and CS/rGO beads over MB and IC, respectively, under flow conditions. Finally, the applicability of mixed CS/GO–CS/rGO beads for the removal of water contaminated with mixed dyes was also demonstrated.

## Graphical abstract

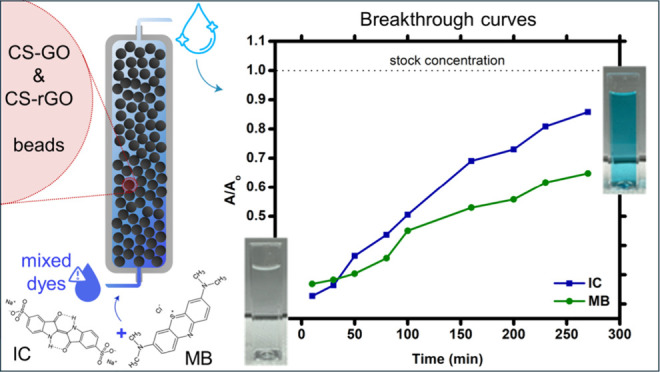

## 1 Introduction

The continuous increase of dyes in aquatic ecosystems is a consequence of human activities, especially the textile industry, which affects the availability of freshwater.^1^ More than 10,000 types of pigments are used in the textile industry, and more than 280,000 tons of textile dyes are discharged into the environment every year. These contaminated effluents have a drastic impact on the ecosystem and cause serious environmental and health problems.^2^ It is important to note that even low concentrations of these dyes can have harmful effects on human health, as these toxic substances enter our food chain through water contamination and then accumulate in living organisms.^3^ Among all other methods to reduce dye contamination, adsorption is one of the most effective and competitive methods due to its economic feasibility, simplicity, and efficiency.^4^ The development of efficient and sustainable adsorbents for water purification is an urgent scientific and technological challenge, especially in view of the growing global demand for clean water and the increasing presence of new pollutants. Among the different classes of adsorbents, aerogels have attracted much attention due to their exceptional porosity, low density, and large specific surface area, which together enable high adsorption capacity. However, translating these outstanding properties into practical applications often requires the development of materials with improved mechanical stability, processability, and ease of handling. On the other hand, the development of adsorbents that exhibit favorable properties for the adsorption toward a wide range of pollutants is another challenging task.^5^

Graphene oxide (GO) is a two-dimensional carbon-based material. It is a promising material as it has a large surface area and various functionalities such as hydroxyl, carbonyl, epoxy, and carboxyl groups, which make it a suitable candidate for various applications ranging from optics to sensors and environmental remediation.^6,7^ The pi−π-conjugated structure of GO can be restored when the oxygen-containing moieties are removed, leading to the so-called reduced graphene oxide (rGO).^8^ When developing advanced materials for the adsorption process, however, it is important to recognize that although nano/microparticles may exhibit excellent adsorption capacities for various pollutants due to their large surface area, their recovery is still a challenge.^9^ Even though GO and rGO exhibit excellent adsorption potential for a wide range of contaminants, the nanoparticle separation from water poses serious difficulties. One solution to this problem that has been frequently proposed in the literature is the incorporation of magnetic nanoparticles into GO or rGO nanosheets, which allow separation via magnetic fields. However, this approach is associated with additional production costs and could lead to metal leaching, which could compromise the efficiency and environmental safety of the composite material.^10^ Porous aerogels, obtained via the freezing of precursor hydrogels, are widely studied soft materials thanks to their elastic nature, high swelling behavior, and micro/nanoporous architecture.^11^ The combination of GO and/or rGO with suitable polymeric materials in the form of aerogels could thus ensure the development of advanced adsorbents that are easy to separate while retaining the possibility of capturing different molecules by utilizing different types of interactions such as electrostatic interactions but also van der Waals or hydrophobic interactions. This represents a key aspect for advanced adsorbents, since various types of dyes frequently coexist in real wastewater, but the removal of mixed pollutants by a single adsorbent is often overlooked when designing new materials. With respect to the huge amount of research dealing with single-dye adsorption, examples of materials exhibiting broad-spectrum removal ability or mixed-pollutant adsorption experiments are less frequent. As an example, Yu et al. proposed cellulose nanofibril/carbon hybrid aerogel monoliths for the adsorption of cationic (methylene blue, MB) and anionic (congo red, CR) dyes. The authors investigated not only the adsorption capacities in single-dye adsorption systems but also binary dye systems.^12^ Similarly, Su et al. reported extremely high adsorption capacity toward both MB and CR in single-dye systems for 3D pomelo-peel cellulose/chitosan/sodium alginate composite aerogel, providing also additional insights into binary dye adsorption behavior.^13^ A 3D monolithic chitosan/graphene oxide (CS/GO) composite aerogel showed high adsorption capacities for methyl orange (543.4 mg/g) and methylene blue (110.9 mg/g), anionic and cationic dyes respectively at 25 °C.^10^ Recently, a κ-carrageenan/polyacrylamide aerogel incorporated with GO nanosheets showed high strength and adsorption capacities (CR: 42.95 mg/g, MB: 105.18 mg/g).^14^ In another study, a low-cost cellulose/activated carbon monolith composite exhibited high uptake of MB and rhodamine B, both individually and from their mixed solutions.^15^

Despite growing interest, the controlled synthesis of aerogel beads with tailored structural and surface properties remains an important research topic. The present work addresses this gap by developing a straightforward and scalable approach to produce adsorbent aerogel beads with improved performance and highlighting their potential impact on advanced water purification technologies. In particular, we prepared nanocomposite chitosan/graphene oxide (CS/GO) and chitosan-reduced graphene oxide (CS/rGO) aerogel beads with sizes in the millimeter range. Previous investigations demonstrated that beads have several advantages over adsorbents in powder form, namely, better stability, reusability, and ease of separation. However, the actual exploitation in fixed-bed columns needs to be optimized to ensure that the adsorbent stay stable and undamaged during operation.^16^ Here, a straightforward three-step procedure that included physical cross-linking, chemical cross-linking, and lyophilization was used to produce aerogel beads. Both physical cross-linking and chemical cross-linking enhance the mechanical properties and stability of the aerogels.^17^ The performance of the synthesized aerogel beads was evaluated by using batch adsorption studies, where both CS/GO and CS/rGO beads were used to adsorb two model dyes with different charges. We have chosen that Indigo carmine (IC, anionic) is widely used in the food, cosmetics, medical, pharmaceutical, and textile industries. Although there are numerous applications for IC, it is highly toxic, causing respiratory, gastrointestinal, and skin irritation.^18^ Due to inefficient fixation methods, about 30% IC is discharged into the environment.^19^ The other dye is methylene blue (MB, cationic), the most common water-soluble basic dye, which is widely used in textile, food, and paper industries and for medical purposes. Despite its applications, inhalation of this dye can make breathing difficult, and ingestion can increase heart rate, cause vomiting, nausea, and eye/skin irritation.^20^ Besides batch adsorption studies, the beads were used as packing material in a fixed-bed column setup to simulate real-world wastewater treatment conditions. The comparative evaluation of batch and dynamic adsorption tests revealed that composite aerogel beads exhibit remarkable efficacy in batch and dynamic systems, highlighting the potential of CS/GO and CS/rGO aerogel beads for water purification.

## 2 Experimental Section

### 2.1 Materials

Chitosan (CS) powder (medium molecular weight, deacetylation degree 75–85%), acetic acid, glutaraldehyde (GA, 25% water solution), and sodium hydroxide pellets were all purchased from Sigma-Aldrich. Indigo carmine (IC) was purchased from Carlo Erba. Methylene blue (MB), graphite flakes (75% over 150 μm, 332461), potassium permanganate (KMnO_4_, 99%), sulfuric acid (H_2_SO_4_, 98%), and hydrogen peroxide (H_2_O_2_, 30%) were purchased from Merck Ltd. Hydrochloric acid (1 M) and sodium hydroxide (0.1 M) solutions for pH adjustments were purchased from VWR Chemicals and Carlo Erba, respectively. Sodium chloride was purchased from Alfa Aesar, and simulated sea salt was purchased from Aquaforest Sea.

### 2.2 Aerogel Bead Preparation

Graphene oxide and reduced graphene oxide nanoparticles were obtained following a low-cost route for top-down synthesis proposed by Trikkaliotis et al.^21,22^ Then, GO or rGO suspension in distilled water (25 mg mL^–1^) was obtained by ultrasonication for 1 h. Chitosan solution was obtained by dissolving CS powder in aqueous acetic acid solution (98/2 vol %/vol %) at a concentration of 25 mg mL^–1^. The nanoparticle suspension (i.e., either GO or rGO aqueous suspension) was gradually added to the polymer solution in a 1:1 ratio. This ratio was chosen so as to get a 1:1 polymer:nanoparticle ratio in the composite beads, since preliminary experiments showed that it allows to get mechanically coherent beads with nanoparticles fully embedded and cross-linked to the macroporous polymeric framework. The system was kept under stirring to get a homogeneous mixture, which was then added dropwise to a 1 M NaOH solution to induce chitosan physical gelation and thus stabilize the bead shape.^23,24^ The obtained nanocomposite hydrogel beads were soaked in an aqueous glutaraldehyde solution (1 mg mL^–1^) for 2 h to induce chemical cross-linking.^25^ Finally, the hydrogel beads were frozen in an acetone bath at −30 °C and freeze-dried. The three-step process for aerogel bead preparation is sketched in Figure 1.

**Figure 1:**
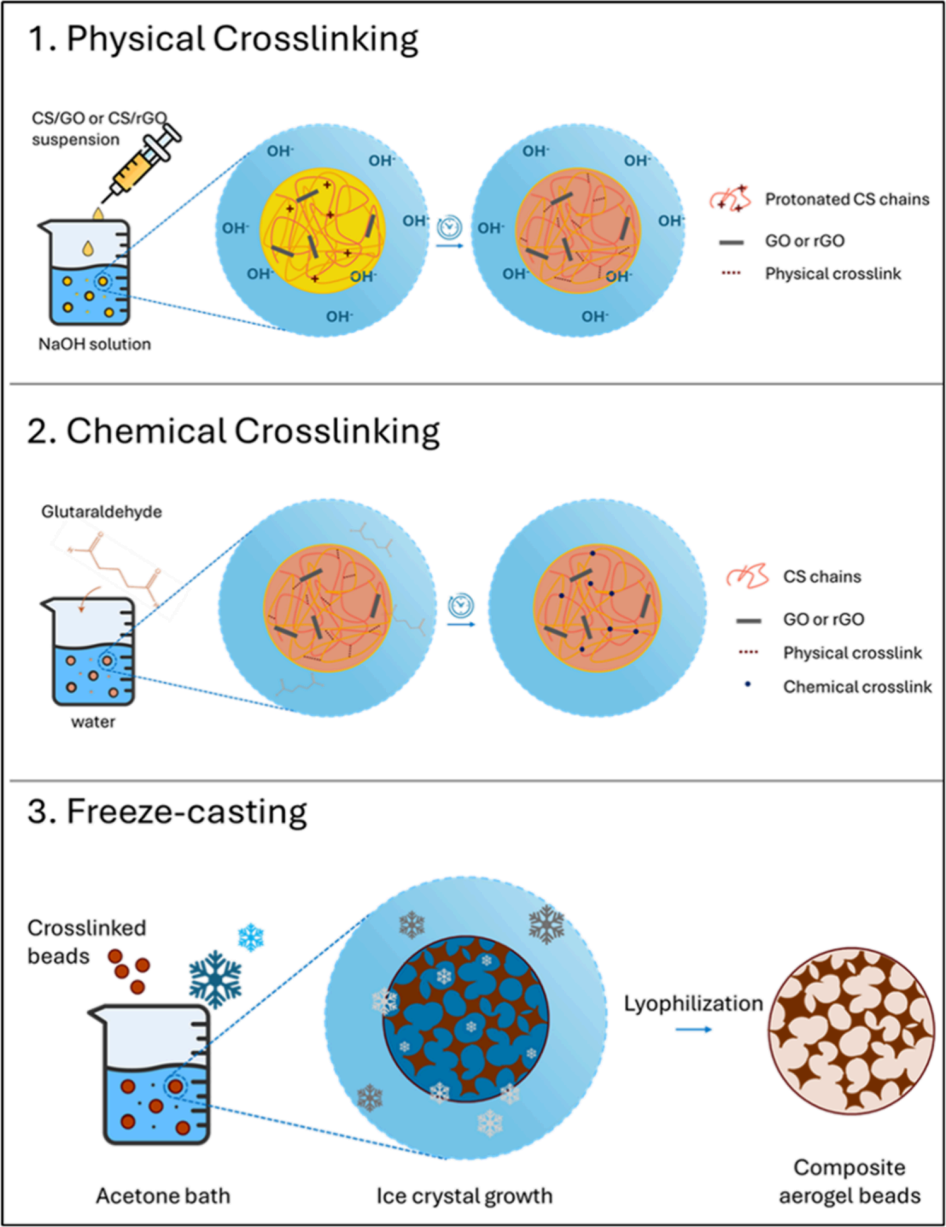
Schematic representation of the three-step production procedure of CS/GO and CS/rGO aerogel beads.

### 2.3 Aerogel Bead Characterization

The size distribution of the aerogel beads was determined by image analysis with ImageJ software.^26^ An equivalent diameter was defined for each bead as the diameter of the circle having the same area as the projection of the bead in the image. The morphology of the nanocomposite aerogel beads was investigated by scanning electron microscopy analysis (SEM, TESCAN VEGA). To image the inner microstructure, the beads were glued to a carrier, held in liquid nitrogen for 1 min, and then cut with a cold razor. Isothermal nitrogen adsorption and desorption tests were carried out at 77 K to measure the surface area and pore size distribution of the samples. Measurements were taken using a commercial volumetric apparatus (Micromeritics 3Flex) on samples previously degassed overnight at 150 °C in a nitrogen stream. The specific area (SSA) was estimated by applying the Brunauer–Emmett–Teller (BET) model to the experimental data. The cumulative pore size and differential pore size distribution were evaluated by using the density functional theory (DFT) slit kernel.

To verify the chemical cross-linking, the aerogel beads (∼100 mg) were soaked in distilled water (30 mL) and placed into a shaker at 25 °C and 120 rpm. At different time intervals (3 and 6 h), the liquid medium was carefully separated and subsequently analyzed by UV/vis spectroscopy to check if nanoparticles/residues are present in water. Then, water absorption capacity, *W*_A_, of nanocomposite aerogels was assessed. The beads (∼5 mg for either CS/GO or CS/rGO) were weighed and subsequently soaked into distilled water. The beads were left at room temperature for 4 days. After the soaking period, the aerogel beads were removed, and excess surface water was eliminated. The water absorption capacity was then calculated as^27^

$$W_{A}=\frac{W_{s}-W_{d}}{W_{d}}$$ 1

where *W*_d_ and *W*_s_ represent the weight of dry and fully swollen aerogels, respectively.

The mechanical stability of the beads was assessed by compression tests carried out at 0.5 mm min^–1^ (Tensometer 2020, Alpha Technologies). The tests were performed on a single layer made of 50 beads, and the resulting curves were rescaled to assess the single-bead mechanical behavior.

The adsorption behavior of aerogel beads was thoroughly investigated by batch tests. Indigo carmine (IC) and methylene blue (MB) dyes were exploited as model pollutant molecules. Kinetic analysis was performed by using an initial dye concentration of 100 mg L^–1^ and an adsorbent/dye solution ratio of 1 g L^–1^, without any pH adjustment. The plastic tubes containing the dye solution and the aerogel beads were shaken at 120 rpm and 25 °C using a thermostated shaker incubator (SKI 4, Argo Lab). The dye concentration was monitored over time up to 900 min, by analyzing the solution with a UV–vis spectrophotometer at the characteristic maximum absorbance wavelength of each dye, namely, 610 nm for IC and 662 nm for MB.^28,29^ Representative UV–vis spectra of the dye solutions, along with the corresponding chemical structures of the dye molecules, are shown in Figure S1 of the Supporting Information. The amount of dye adsorbed over time, *q*_*t*_, was calculated as

$$q_{t}=\frac{V}{m}\left(C_{0}-C_{t}\right)$$ 2

where *C*_0_ is the initial dye concentration (mg L^–1^), *C_t_* is the dye concentration at time *t* (mg L^–1^), *V* is the volume of the dye solution (L), and *m* is the adsorbent mass (g).

Once the time needed to attain equilibrium conditions was determined, adsorption isotherms were performed by varying the initial dye concentration. The equilibrium adsorption capacity, *q*_e_, was calculated as

$$q_{e}=\frac{V}{m}(C_{0}-C_{\mathrm{eq}})$$ 3

where *C*_eq_ is the dye concentration at equilibrium (mg L^–1^).

To complete the picture on adsorption behavior, the effect of pH and interfering ions was investigated. More specifically, the pH of distilled water (pH = 6.75) was varied in the range 2–12 by adding proper amounts of 1 M HCl and 0.1 M NaOH solutions. As interfering ions, 3.5 wt/vol % NaCl and 3.8 wt/vol % of simulated sea salt were considered.

Besides testing CS/GO and CS/rGO beads separately, mixed beads at different weight ratios (CS/GO:CS/rGO beads = 1:3, 1:1, 3:1) were also used as adsorbent materials for kinetics and isotherm analyses.

A custom-made fixed-bed configuration at a 15 mL scale was exploited for evaluating the effectiveness of the nanocomposite aerogel beads under flow conditions. Details on the experimental apparatus and procedure are given in the Supporting Information (Video S1). The column was filled with a fixed amount of beads (∼1.5 g), and the dye solution (fixed upstream concentration: *C*_upstream_ = 3.5 mg L^–1^) was fluxed at 6 mL min^–1^ by means of a peristaltic pump. The downstream dye concentration, *C*_downstream_, was monitored over time.

## 3 Results and Discussion

### 3.1 Microstructural and Physical Characterization of the Aerogel Beads

The preparation protocol outlined in Figure 1 resulted in almost spherical aerogel beads (circularity ≥0.8) with dimensions in the millimeter scale (Figure 2). In detail, the CS/GO beads are characterized by a relatively narrow size distribution with an average equivalent diameter of 2.84 mm (standard error: 0.06 mm). For the CS/rGO samples, the size distribution is somewhat wider and is characterized by an average equivalent diameter of 3.01 mm (standard error: 0.08 mm). Overall, no statistical differences were found between the average size of the two families of aerogel beads (*p* > 0.05), indicating that the different nature of the nanoparticles does not significantly affect bead formation and the drying process.

**Figure 2:**
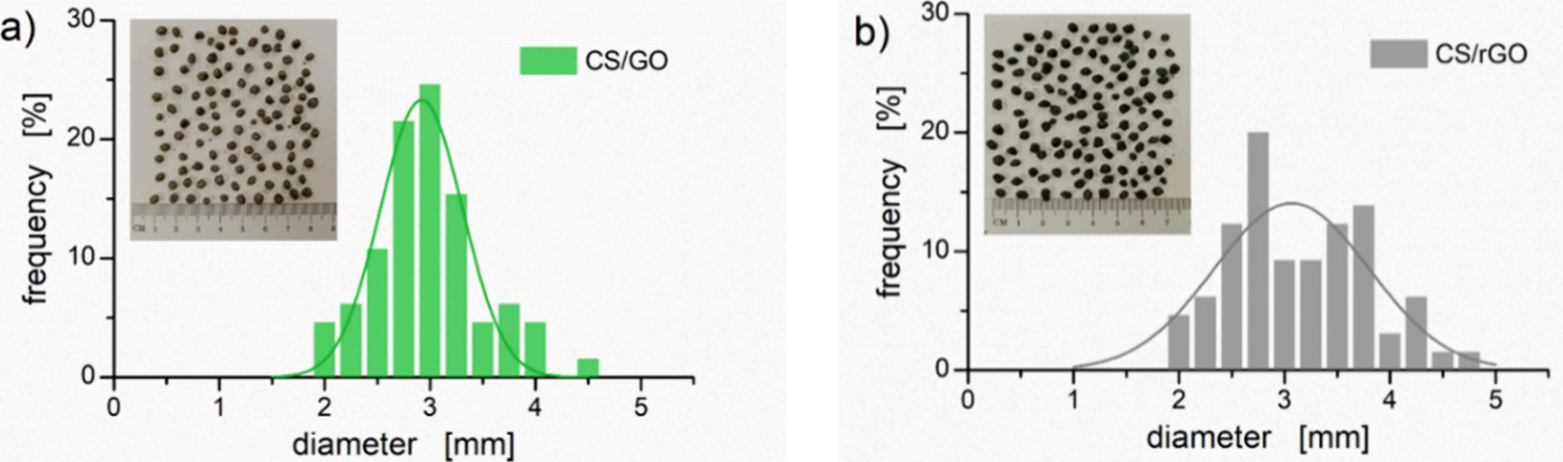
Average size distribution of nanocomposite aerogel beads: (a) CS/GO and (b) CS/rGO. Solid lines are a guide for the eye. Representative pictures of the beads are shown in the inset.

At the micrometer scale, the beads appear quite compact on the outer surface, as shown in the SEM images presented in Figure 3. This is likely due to the polymer/particle skin that forms at the beginning of the physical cross-linking step when the pH gradient at the interface between the colloidal solution droplet and the surrounding alkaline bath is the highest. On the other hand, the interior of the beads is highly porous thanks to the final freeze-drying step. The inner bead microstructure, which is made of a network of highly interconnected pore channels separated by thin filaments/walls, is expected to be beneficial for the full exploitation of GO and rGO surface properties for adsorption.

**Figure 3:**
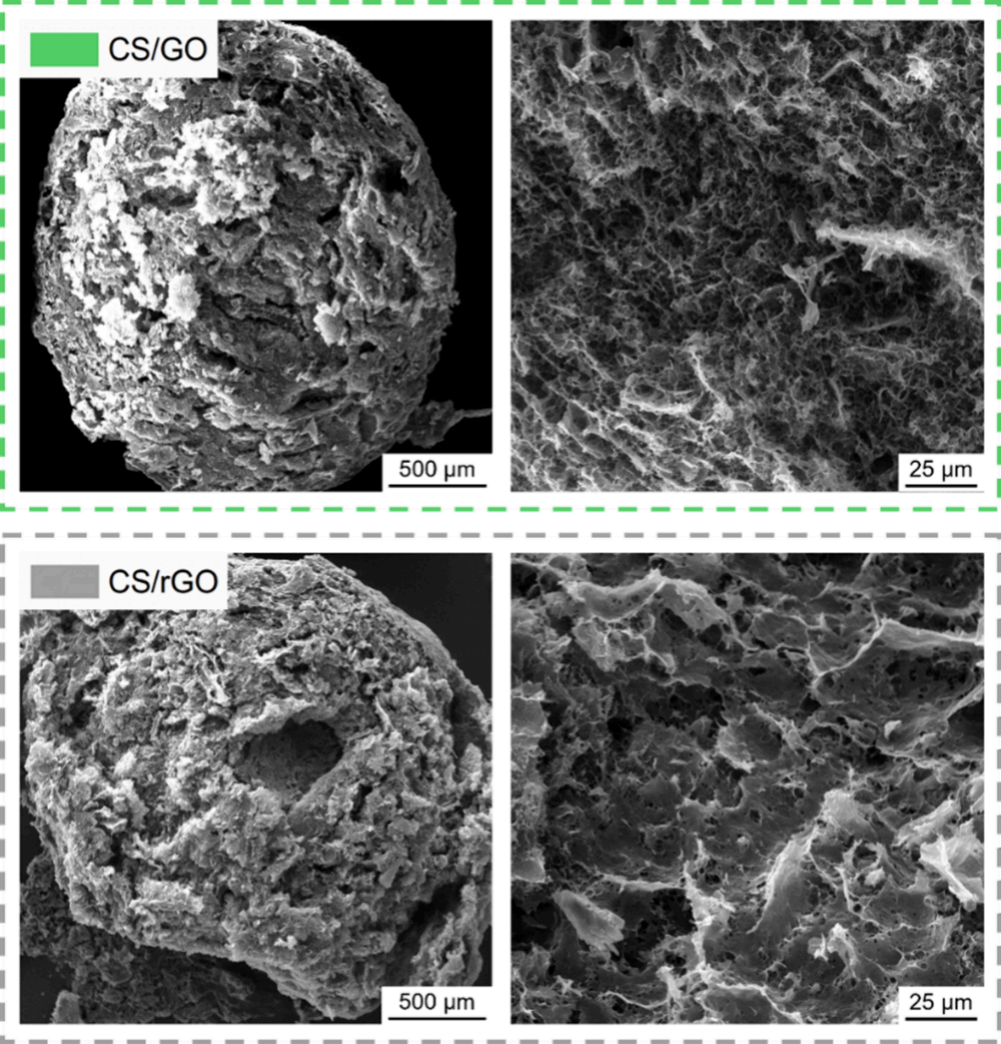
SEM micrographs of nanocomposite CS/GO and CS/rGO aerogel beads. The images on the right-hand side show the interior of cryo-cut beads.

Quantitative information about the porosity of the samples is provided by the isothermal nitrogen adsorption and desorption curves, as shown in Figure 4a. Both samples exhibit a similar type IV isotherm, with a hysteresis loop which is characteristic of macroporous mesoporous materials. Similar features have been already observed in composite aerogels obtained by freeze-drying.^30^ Using DFT analysis, three broad families of pores within the mesopore range (20–500 Å) have been identified (Figure 4b), highlighting the heterogeneity in the morphology of the beads, as also demonstrated by SEM micrographs. Although the pore size distributions of the samples are similar, the addition of GO or rGO differently impacts the morphology of the composites. As a matter of fact, the reduction of GO to rGO is known to cause an exfoliation of the graphene sheets,^31–33^ which results in variations in surface area (34.46 ± 0.15 m^2^ g^–1^ for CS/GO versus 86.05 ± 0.38 m^2^ g^–1^ for CS/rGO) and pore volume (0.018 cm^3^ g^–1^ for CS/GO versus 0.041 cm^3^ g^–1^ for CS/rGO). The enlargement of the size of the pores, together with the high surface area, could have a positive effect on the diffusion of large dye molecules inside the aerogels.

**Figure 4:**
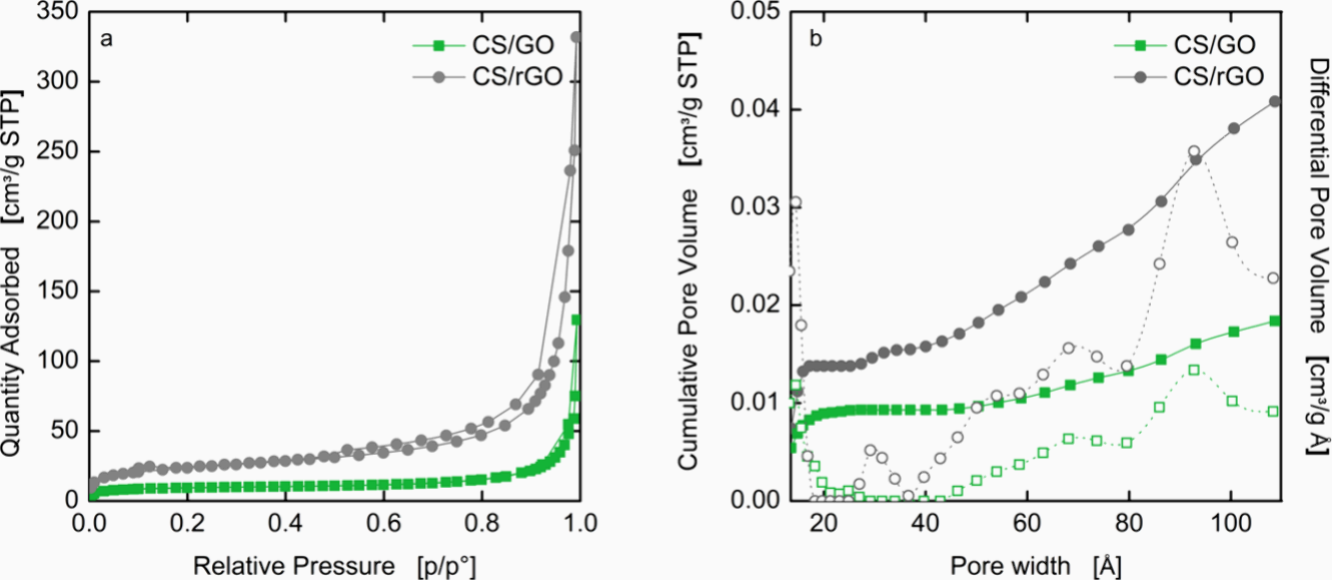
(a) N_2_ adsorption–desorption isotherms and (b) pore volume analysis: full and open symbols correspond the cumulative and differential pore volume distribution, respectively.

Despite the highly porous structure, the aerogel beads possess good mechanical stability. Representative compressive stress–strain curves are shown in Figure 5. The investigated aerogel beads show the typical behavior of porous material: after an initial phase of elastic deformation, followed by a plateau region corresponding to the collapse of the pores, the material undergoes a densification process in which the stress values increase significantly as the compression progresses.^17,24^ The mechanical resistance of CS/GO aerogel beads is slightly higher than that of CS/rGO ones. This can be ascribed to the higher affinity between the biopolymer and the nanoparticles, which may result in a more homogeneous nanocomposite. Overall, the produced aerogel beads appear to have significant mechanical stability: as a reference value, a single bead, whose weight is roughly 1.5 mg, can withstand an applied load of ∼2 g for CS/GO and ∼1 g for CS/rGO with only 20% of deformation. In addition to the mechanical stability of the dry beads, the stability of the aerogels in water was also assessed. Swelling tests proved that the beads have a high affinity for water (water absorption capacities of 2390 and 2070% for CS/GO and CS/rGO beads, respectively), which is expected to help the dye solution to penetrate the porous microstructure so that the adsorption process can take place. Nonetheless, swelled beads preserve their overall shape after drying and, more importantly, nanoparticles do not leak in water, proving successful cross-linking among chitosan macromolecules and nanoparticles (see Figure S2 of the Supporting Information).

**Figure 5:**
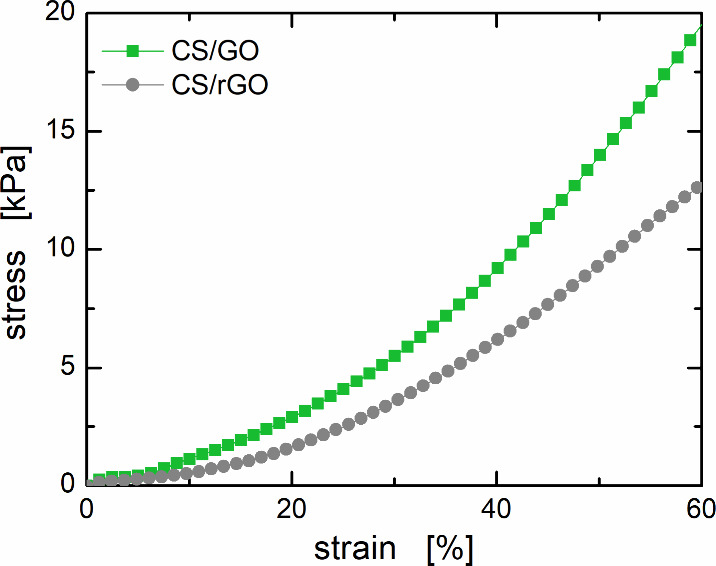
Stress–strain curves of CS/GO and CS/rGO aerogel beads under unconfined uniaxial compression.

FTIR analyses were carried out on dry beads before and after dye adsorption (Figure 6) to gain insights into the adsorbent–dye interaction. The FITR spectra of as-prepared CS/GO and CS/rGO aerogels show the typical peaks and bands of CS, GO, and rGO. In particular, the stretching vibration of the amine group of CS^34^ and OH groups of GO and rGO are visible in the range of 3000–3500 cm^–1^. Moreover, the peaks at around 1230 cm^–1^ can be addressed to bending and stretching vibrations of C–O^35^ while those at 2920, 1150, and 1065 cm^–1^ reflect CH_3_ symmetric stretching, C–O–C stretching, and C–OH stretching of CS groups, respectively.^34^ After the adsorption experiments, the aerogels were collected and dried. Both CS/GO and CS/rGO beads show a shift in the band at 3000–3500 cm^–1^, suggesting that OH groups of GO and rGO are involved in the dye uptake process. Nonetheless, more relevant differences can be noticed in the spectra of CS/GO after the adsorption of MB and CS/rGO after the adsorption of IC, further confirming their enhanced affinity versus an anionic or cationic dye, respectively. As an example, a clear shift in the peak at 1230 cm^–1^ of CS/GO can be detected, together with the appearance of the peak at 1485 cm^–1^, which is representative of the C=S vibration of MB.^35^ Similarly, the peak at 1100 cm^–1^, representative of the stretching of S=O, appears in the CS/rGO spectrum after adsorption of IC,^36^ while a shift in the peak at 1415 cm^–1^ and the appearance of a peak at 1610 cm^–1^, characteristic of benzene ring stretching, is detected.^35^

**Figure 6:**
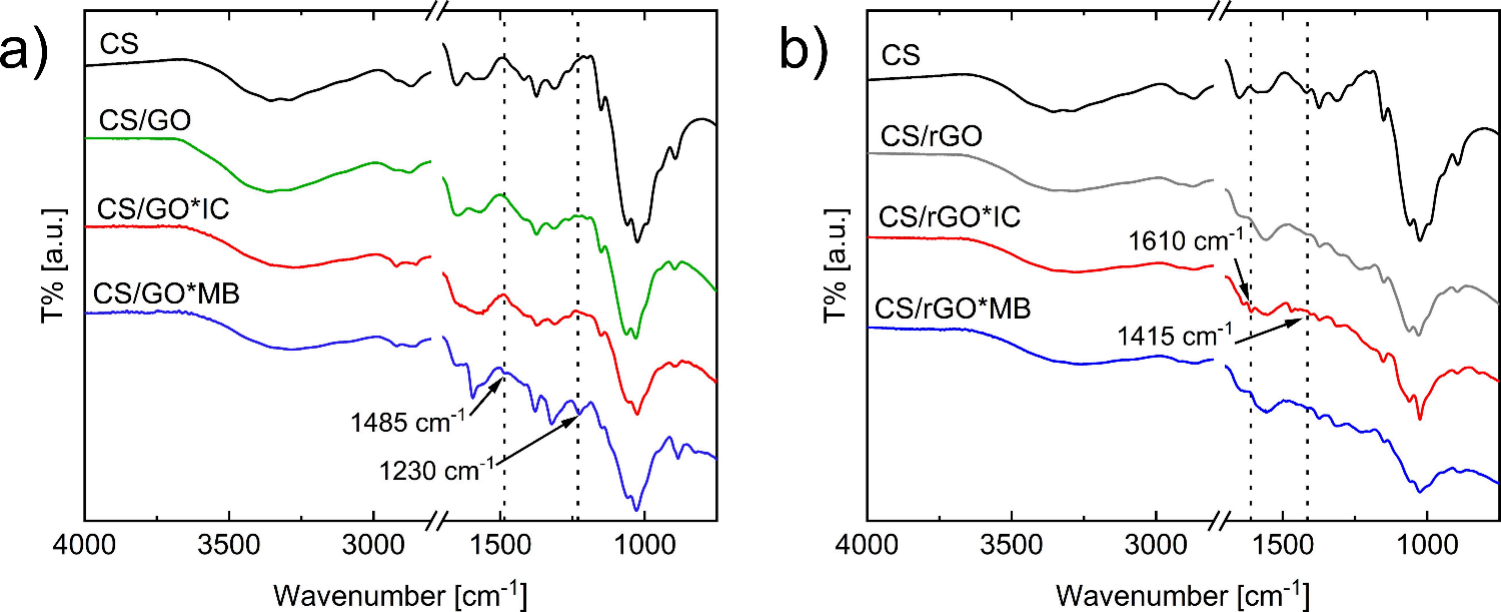
FTIR spectra of as-prepared (a) CS/GO and (b) CS/rGO beads. Labels “*IC” and “*MB” indicate beads after adsorption tests. Adsorption experiments were performed with an initial dye concentration of 100 mg L^–1^, an adsorbent/dye solution ratio of 1 g L^–1^, pH = 6.7, for IC and 6.8 for MB, and 25 °C. Spectrum of pristine CS is reported as reference.

### 3.2 Effect of pH

Additional insights into the adsorption mechanisms can be gained by looking at the surface charge of the nanocomposite aerogel beads (Figure 7c). It was found to be quite similar, with points of zero charge of 7.28 and 6.88 for CS/GO and CS/rGO beads, respectively. It thus emerges that optimal adsorption conditions can be achieved by adjusting the pH toward either acidic or alkaline environments, depending on the ionic nature of the dyes. However, since the adsorption mechanism also involves other types of interactions—such as π–π stacking between the aromatic rings of GO/rGO and those of the dyes—a more detailed analysis of the pH effect on adsorption has been carried out. The results are reported in Figure 7. In strongly acidic conditions (pH ∼ 2), the adsorption sites of CS/GO beads are protonated, leading to a slight increase in the adsorption of anionic species (here IC dye). On the other hand, at high pH values, although the increased negative surface charge is detrimental for adsorbent–dye electrostatic interactions, the adsorption process can still rely on strong hydrogen bonding and π–π interactions. For CS/rGO, instead, the adsorption of IC is dramatically high because of the increase in the hydrophobic character and setting up of π–π interactions.^37^ For what concerns MB, the enhanced negative character of CS/rGO beads at high pH further improves adsorption through strengthened electrostatic interactions with the cationic MB^+^ species.^27^ Under acidic conditions, a high proton concentration neutralizes MB and decreases the electrostatic attraction. As a result, CS/GO and even more CS/rGO showed higher MB^+^ adsorption in alkaline rather than acidic conditions.^38^

**Figure 7:**
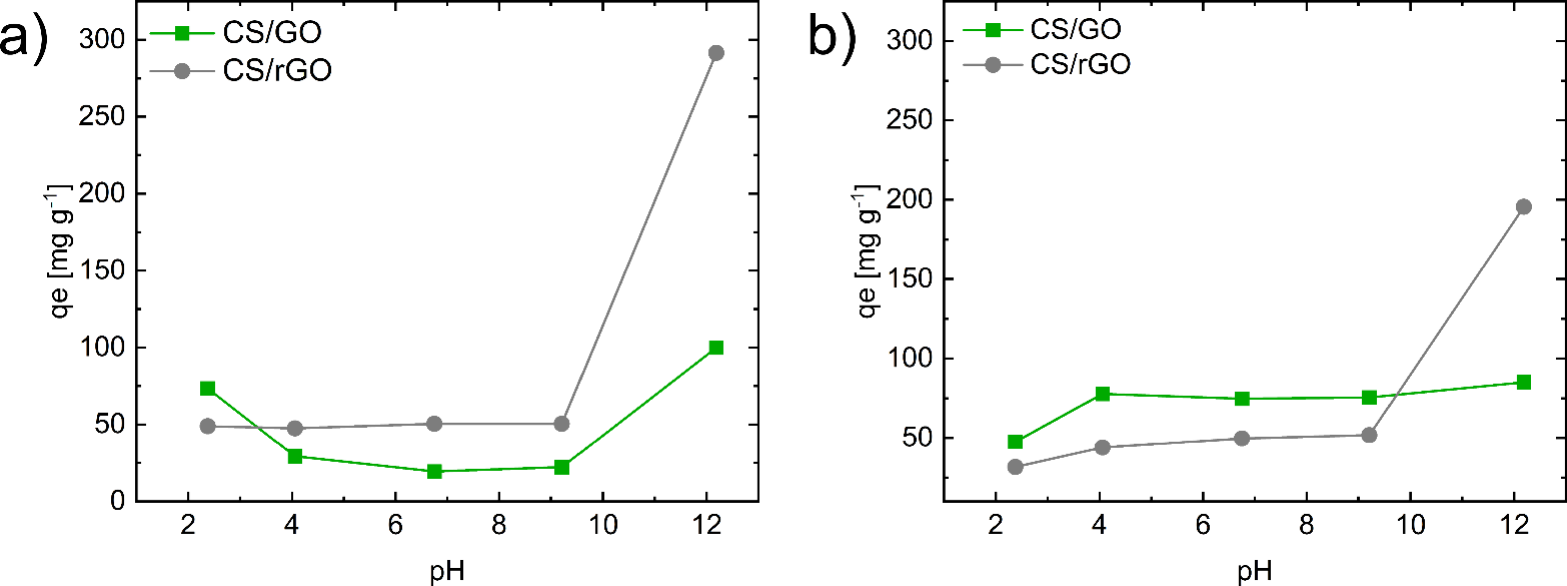
Effect of pH on the adsorption performance of CS/GO and CS/rGO beads for (a) IC and (b) MB. Experiments were performed with an initial dye concentration of 100 mg L^–1^, adsorbent/dye solution ratio of 1 g L^–1^, and 25 °C.

### 3.3 Adsorption Mechanism

For IC, the optimal pH value was found to be 12. According to Figure 8a, at this level, both adsorbents are negatively charged (pH > pH_zpc_), while IC is positively charged (p*K*_a_ 12.2).^39^ Thus, it is suggested that the main adsorption mechanism involves electrostatic interactions. However, there are also other minor forces that enhance adsorption, as previously described, including π–π and *n*–π interactions and H-bonding. In the case of MB (p*K*_a_ 3.8),^40^ adsorbents are negatively charged at pH 12. MB being a cationic dye indicates that the adsorption mechanism depends on electrostatic interactions, but also on weaker forces like hydrophobic interactions, π–π and *n*–π interactions, and H-bonding and Yoshida H-bonding.^41^ Figure 8b schematically shows the interactions that mainly govern the adsorption process for the two types of nanocomposite aerogels.

**Figure 8:**
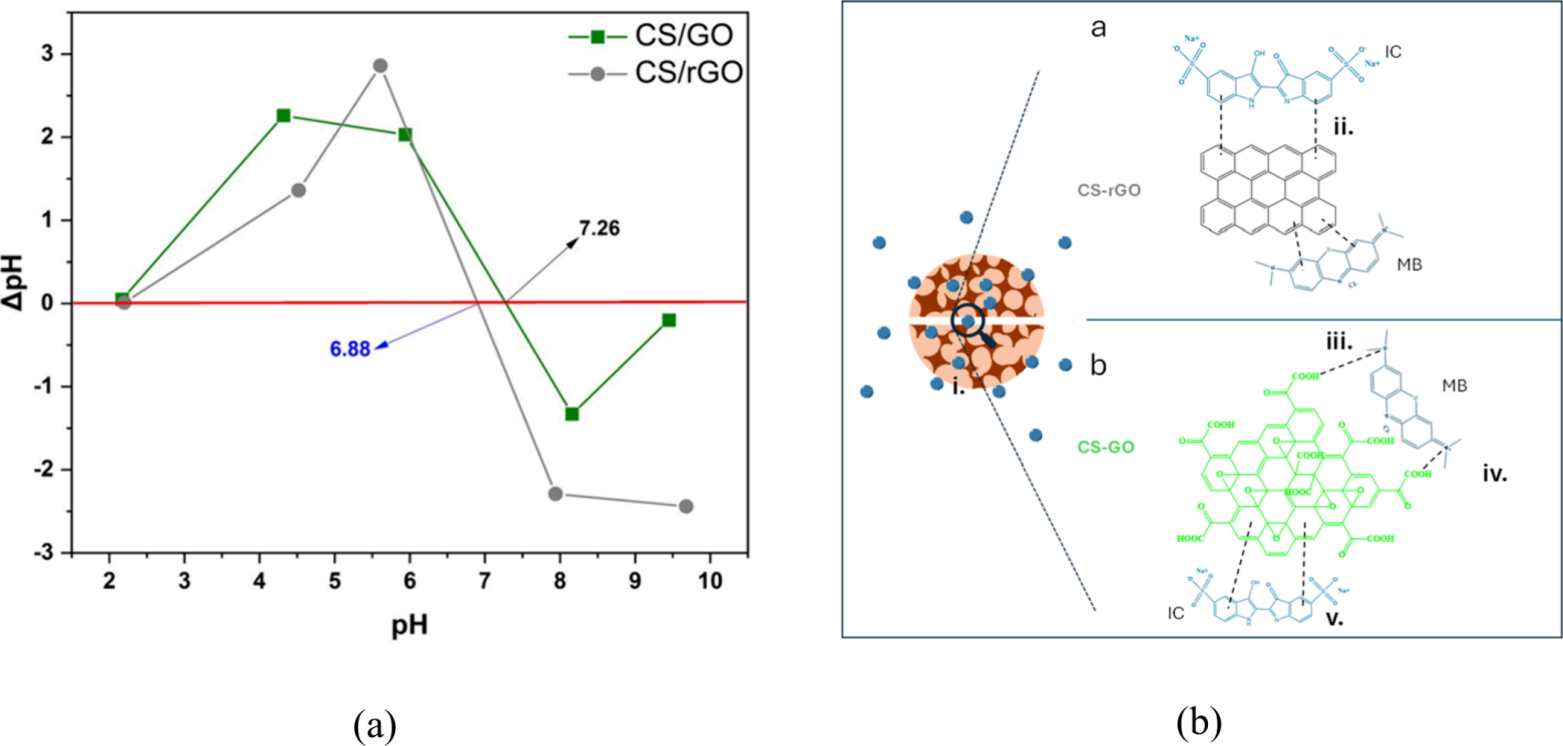
(a) Calculation of pH_zpc_ of adsorbents by using pH change according to the drift method^42^ and (b) proposed adsorption mechanism of IC and MB on (a) CS/rGO and (b) CS/GO aerogel beads. (i) Physical adsorption or pore diffusion, (ii and v) π–π interactions, (iii) hydrogen binding, and (iv) electrostatic interactions.

### 3.4 Effect of Contact Time—Kinetics

After the main adsorbent–dye interaction mechanism was identified, the adsorption process was investigated in terms of kinetics and isotherms by batch testing with dye solutions without any pH adjustment. The results of kinetics tests are reported in Figure 9, which shows the amount of dye adsorbed over time calculated according to eq 2.

**Figure 9:**
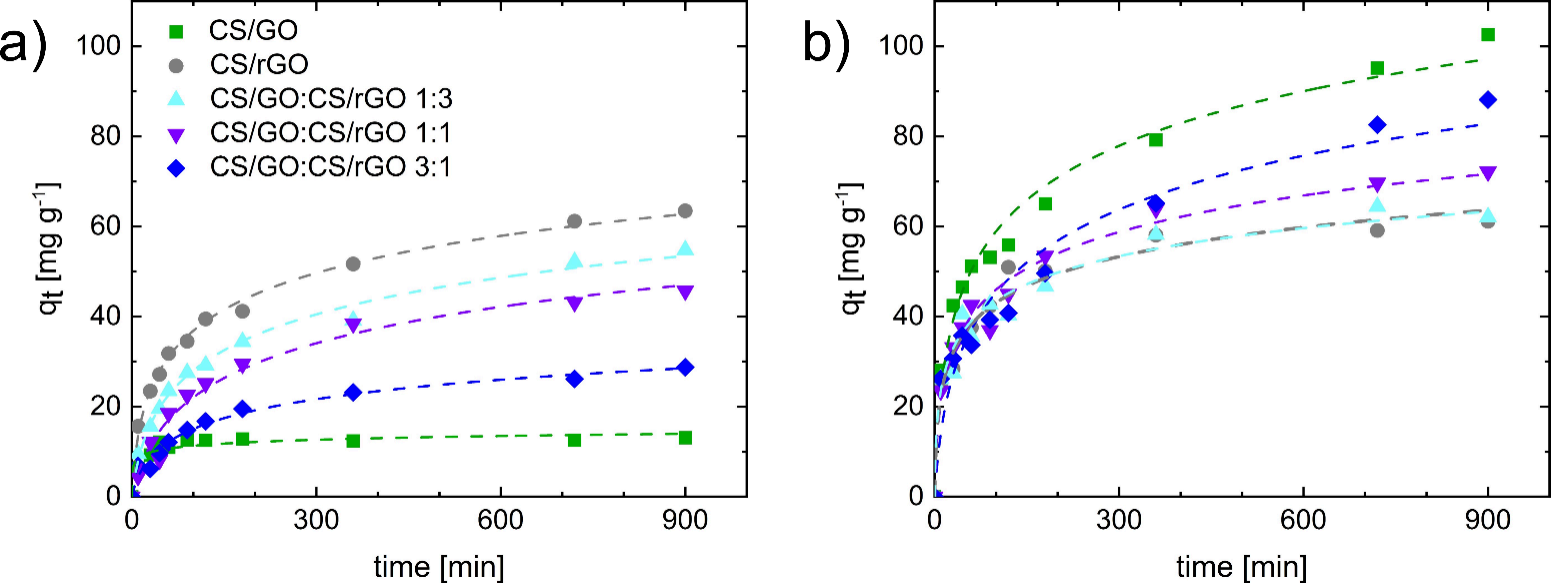
Adsorption kinetics for (a) IC and (b) MB dyes. Dashed lines correspond to best fitting to the Elovich kinetic model (eq 4). Experiments were performed with an initial dye concentration of 100 mg L^–1^, an adsorbent/dye solution ratio of 1 g L^–1^, pH = 6.7 for IC and 6.8 for MB, and 25 °C.

From the first analysis of adsorption kinetics, optimal contact times that were then adopted for equilibrium adsorption tests were set for CS/GO beads to ∼6 and ∼20 h for IC and MB, respectively, while ∼15 and ∼10 h were considered for CS/rGO beads. Going more in detail, the experimental data was fitted to the pseudo-first order (PFO), pseudo-second order (PSO), Elovich, and intraparticle diffusion kinetic models (reported as eqs 4–7, respectively).^43,44^

$$q_{t}=q_{e}(1-r^{-k_{1}t})$$ 4

$$q_{t}=\frac{q_{e}^{2}k_{2}t}{1+q_{e}k_{2}t}$$ 5

$$q_{t}=\frac{1}{\beta }\ln\left(1+\alpha \beta t\right)$$ 6

$$q_{t}=k_{\mathrm{ip}}\sqrt{t}+c_{\mathrm{ip}}$$ 7

where *k*_1_ and *k*_2_ are rate constants for the PFO and PSO kinetic models, respectively, α and β represent the initial adsorption rate and the desorption constant according to the Elovich model, and *k*_ip_ and *c*_ip_ correspond to the coefficients of the intraparticle diffusion model.

The statistical parameters indicate that the kinetic data in Figure 9 are best described by the Elovich model in most of the adsorbent–dye combinations (fitting parameters are reported in Tables S1–S4 in the Supporting Information), suggesting the presence of heterogeneous surfaces with different adsorption energies. The gradual decrease in adsorption rate over time indicate a progressive occupation of active sites, where interactions between the adsorbate and adsorbent become less effective as coverage increases.^45^ This result is consistent with the findings on other nanocomposites.^46^ In agreement with previous results on adsorbent–dye affinity, the kinetic tests clearly indicate that CS/GO aerogels have a high ability to remove MB, while their affinity for IC is rather low. In contrast, CS/rGO aerogels have a good adsorption capacity for IC, which is comparable to that for MB (although lower than that of CS/GO). This trend is also in line with previous results from the literature on other GO- and rGO-based composite or hybrid materials.^47,48^

### 3.5 Isotherms

The different aerogel–dye affinity clearly emerges also from adsorption isotherms. Figure 10 shows the amount of dye adsorbed as a function of the equilibrium concentration calculated according to eq 3.

**Figure 10:**
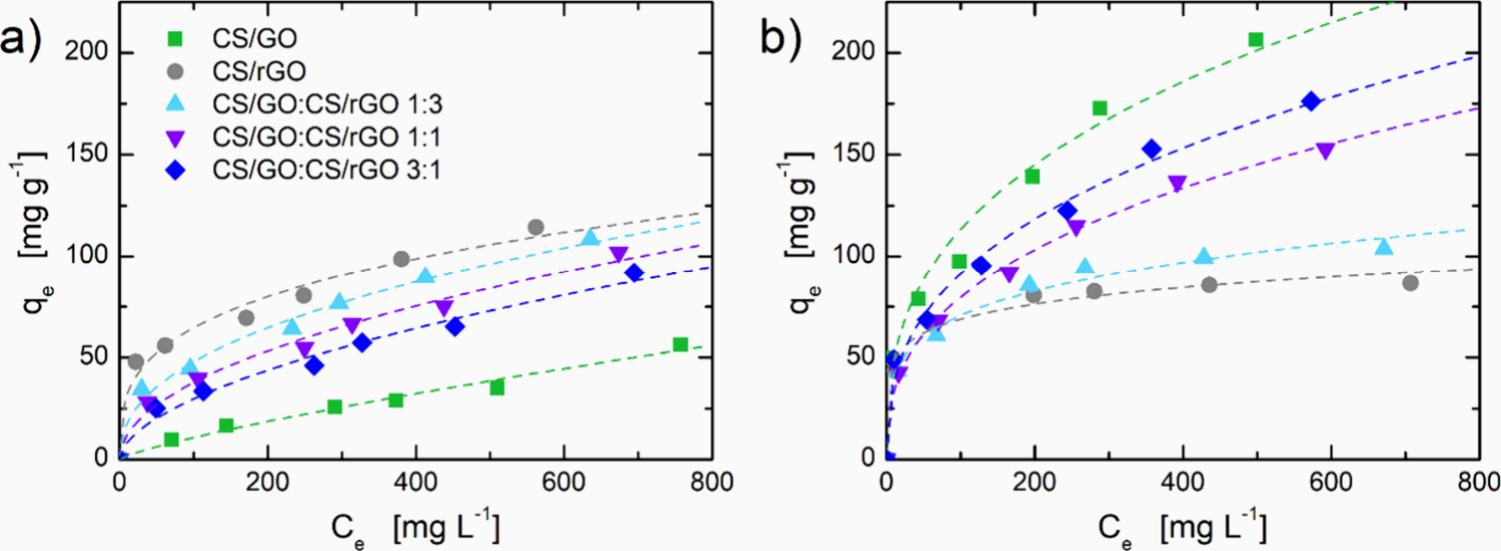
Adsorption isotherms for (a) IC and (b) MB dyes. Dashed lines correspond to best fitting with the Freundlich model (eq 9). Experiments were performed with an initial dye concentration in the range 100–1000 mg L^–1^, adsorbent/dye solution ratio of 1 g L^–1^, pH = 6.7 for IC and 6.8 for MB, and 25 °C.

The two-parameter Langmuir,^49^ Freundlich,^50^ and Temkin^51^ models (reported as eqs 8–10, respectively) and the three-parameter Sips model^52,53^ (eq 11) were fitted to the experimental data:

$$q_{e}=q_{L}\frac{\left(k_{L}C_{e}\right)}{1+k_{L}C_{e}}$$ 8

$$q_{e}=k_{F}C_{e}^{1/n_{F}}$$ 9

$$q_{e}=\frac{RT}{b_{T}}\ln\;k_{T}C_{e}$$ 10

$$q_{e}=q_{s}\frac{(k_{s}C_{e}{)}^{1/n_{s}}}{1+(k_{s}C_{e}{)}^{1/n_{s}}}$$ 11

where *k*_L_ is the Langmuir constant that is related to adsorbent/adsorbate affinity and *q*_L_ represents the maximum adsorption capacity at saturation according to the Langmuir model; *k*_F_ is the Freundlich constant that is related to the adsorption capacity, and *n*_F_ is a parameter that is related to adsorbent/adsorbate affinity; *R* is the universal gas constant, *T* is the temperature, *b*_T_ represents the heat of adsorption, and *k*_T_ is the Temkin isotherm constant; and *q*_s_ is the adsorption capacity a saturation according to Sips model, *k*_s_ is the affinity constant, and *n*_s_ gives a measure of the system heterogeneity (for *n*_s_ = 1 Langmuir model is recovered).^51,54^

For both IC and MB adsorption, the statistical parameters indicate that the experimental data in Figure 9 are best fitted by the Freundlich model (fitting parameters are reported in Tables S5 and S8 in the Supporting Information). Actually, all the adsorption isotherms did not reach a clear saturation plateau, which is consistent with the heterogeneous adsorption process underlying the Freundlich model. Moreover, the fitting procedure yielded 0 < 1/*n*_F_ < 1 for all the adsorbent/adsorbate pairs, indicating that adsorption is a favorable process in all investigated cases.^51^ In terms of adsorption capacity, isotherms further corroborate what has already emerged from a kinetic study. Namely, CS/GO aerogels were found to have a higher adsorption capacity for the cationic dye, while CS/rGO showed a higher removal ability with the anionic dye. As previously evidenced by data in Figure 7, the adsorption mechanism of IC and MB dyes on CS/GO and CS/rGO beads is pH reliant. At acidic pH levels, protonated sites on the CS/GO composites favor IC uptake through electrostatic attraction. However, at basic pH values, π–π interactions and hydrogen bonding dominate adsorption mechanisms, specifically onto the CS/rGO beads, owing to their hydrophobic and graphitic character. For cationic MB, adsorption enhances at high pH as a result of electrostatic attraction, particularly with the CS/rGO composites (see also Figure 8 for the proposed adsorption mechanism).

More quantitatively, according to the *Q*_max_ parameter of the Langmuir model as indication of adsorption capacity, the maximum IC uptake is 109 mg g^–1^ for CS/rGO, while the maximum MB uptake is 254 mg g^–1^ for CS/GO. Evidently, when mixing the two types of beads in different proportions, an intermediate behavior between that of CS/GO and CS/rGO aerogels is observed, allowing to tailor the overall adsorption capacity of the material.

### 3.6 Stability and Regeneration

The stability of the aerogel beads was investigated by soaking both adsorbents in water for 1 week (Figure 11a–g). The results showed that both of them retained their structural integrity and had no deformation in their shape. In addition, the sample stability is confirmed by Figure S2, showing the UV–vis spectra of water samples soaking the beads for 6 h. The collected water is essentially clean, giving evidence of successful cross-linking (i.e., no nanoparticles leakage from swollen beads).

**Figure 11:**
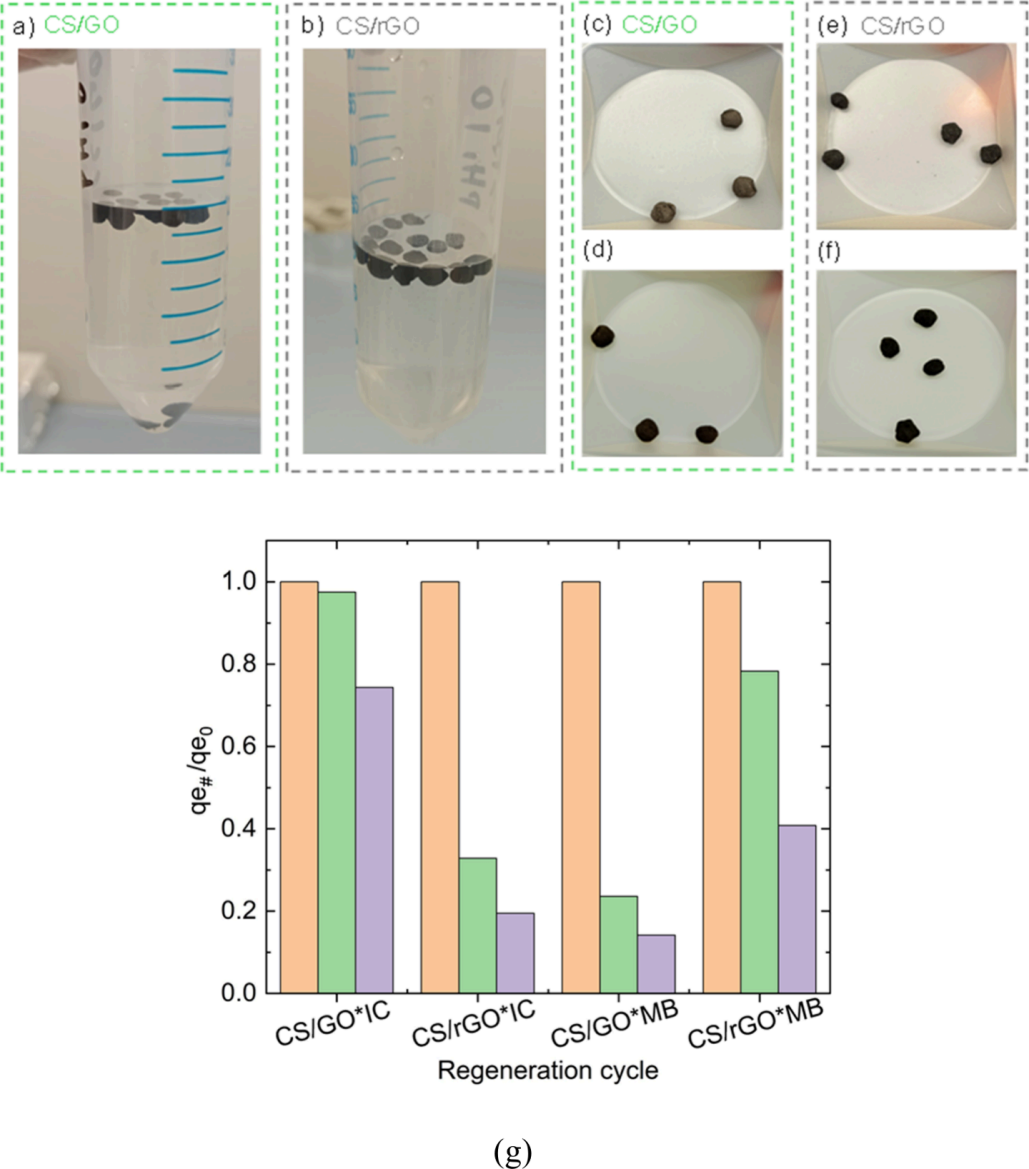
Images of (a) CS/GO and (b) CS/rGO aerogel beads after 1 week in water. Images of dried beads before and after water absorption tests: (c, d) CS/GO and (e, f) CS/rGO. (g) Equilibrium adsorption capacity at cycle # normalized over that at cycle 0. Regeneration adsorption experiments were performed with an initial dye concentration of 100 mg L^–1^, adsorbent/dye solution ratio of 1 g L^–1^, pH 6.7 for IC and 6.8 for MB, and 25 °C.

The regeneration of adsorbents is a crucial factor for their practical application. For this reason, the reusability of aerogel beads was investigated (Figure 11g). For the regeneration studies, a 1 M NaOH solution was prepared and used to induce desorption of the previously adsorbed dyes from the aerogel beads. The regeneration cycles of aerogel beads involved the washing of beads with alkaline solution and then with distilled water three times. The results showed that the adsorption capacity of the beads is preserved after two consecutive cycles. Interestingly, CS/GO beads, which were found to exhibit higher adsorption capacity for MB, exhibit higher stability of the adsorption properties over regeneration cycles in the case of IC. The opposite trend was found for CS/rGO, suggesting that the higher the adsorbent–dye affinity, the lower the regeneration capacity.

### 3.7 Effect of Salts

As final consideration, the adsorption ability of CS/GO and CS/rGO aerogels in the presence of interfering ions has also been investigated to get information about the stability of adsorption performance in real environments. For this purpose, aqueous solution with 3.5 wt/vol % NaCl or 3.8 wt/vol % of simulated sea salt was employed as medium for adsorption tests. As shown in Figure 12a, the adsorption capacity of CS/GO nanocomposites for IC remains unaffected in the presence of simulated sea salts, while a slight increase is observed in the presence of 3.5 wt/vol % NaCl, suggesting no significant interference of anionic species.^55^ CS/rGO beads, instead, showed a reduction in IC adsorption in the presence of 3.5 wt/vol % NaCl. Adsorption of MB by CS/rGO is also in part reduced by the presence of salts (Figure 12b), indicating an interfering effect of ionic species in the main adsorption mechanisms of CS/rGO aerogels, that is, pi–pi interactions. Conversely, adsorption of MB onto CS/GO is markedly influenced by salt addition, which evidently competes with positive dye molecules leading to a reduction in adsorption capacity.^56^

**Figure 12:**
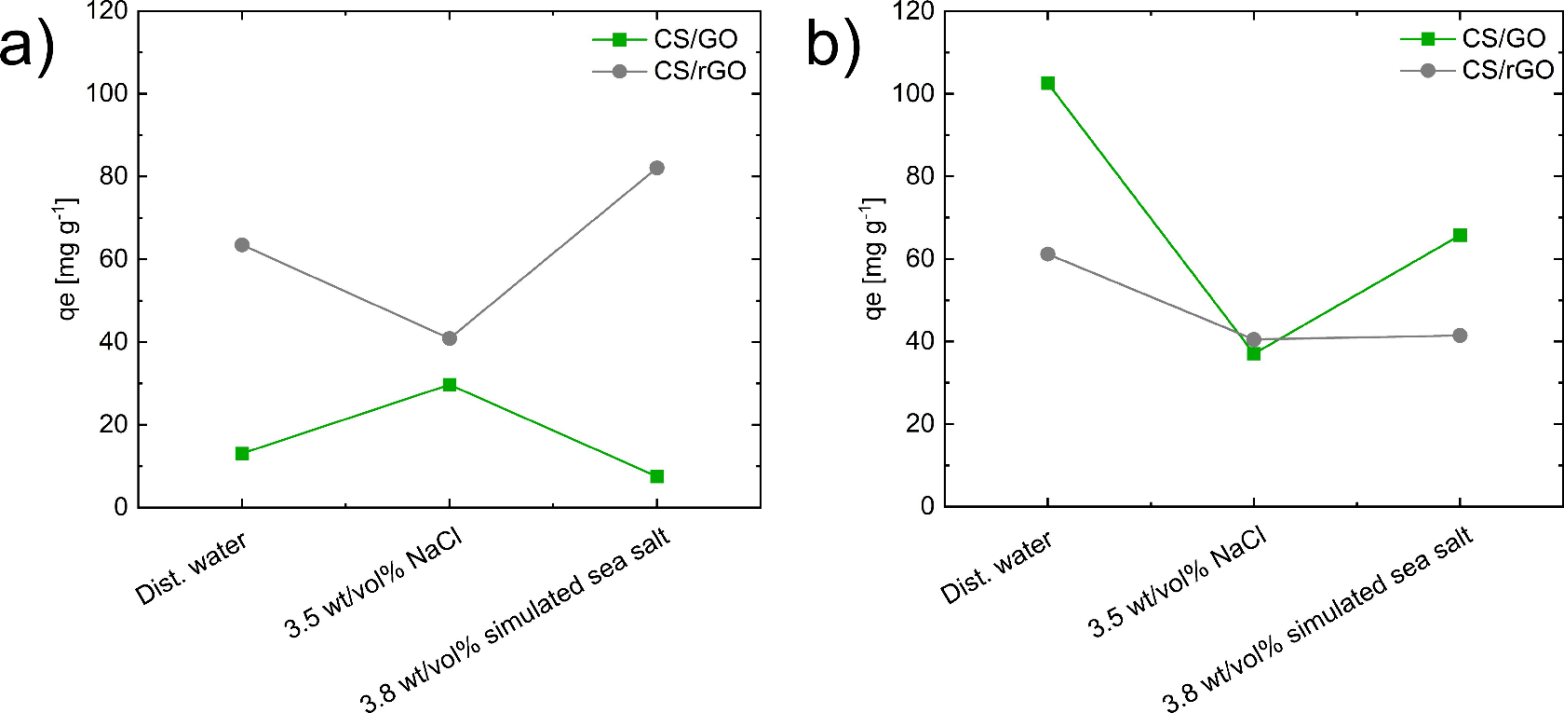
Effect of coexisting ions on the adsorption performance of CS/GO and CS/rGO for (a) IC and (b) MB. Experiments performed with an initial dye concentration of 100 mg L^–1^, adsorbent/dye solution ratio of 1 g L^–1^, pH = 6.7 for IC and 6.8 for MB, and 25 °C.

### 3.8 Adsorption Properties: Column Tests

A small-scale flow test setup has been conceived and exploited to simulate operating fixed-bed conditions (details in Section 2 and in Video S1). Based on the results of batch adsorption tests, CS/GO aerogel beads were selected for MB removal in a fixed-bed adsorption column, while CS/rGO aerogel beads were used as packing material for IC adsorption. The evolution of the dye concentration once the contaminated water passed through the column filled with the aerogel beads was monitored over time, and the results obtained are shown in Figure 13a,b.

**Figure 13:**
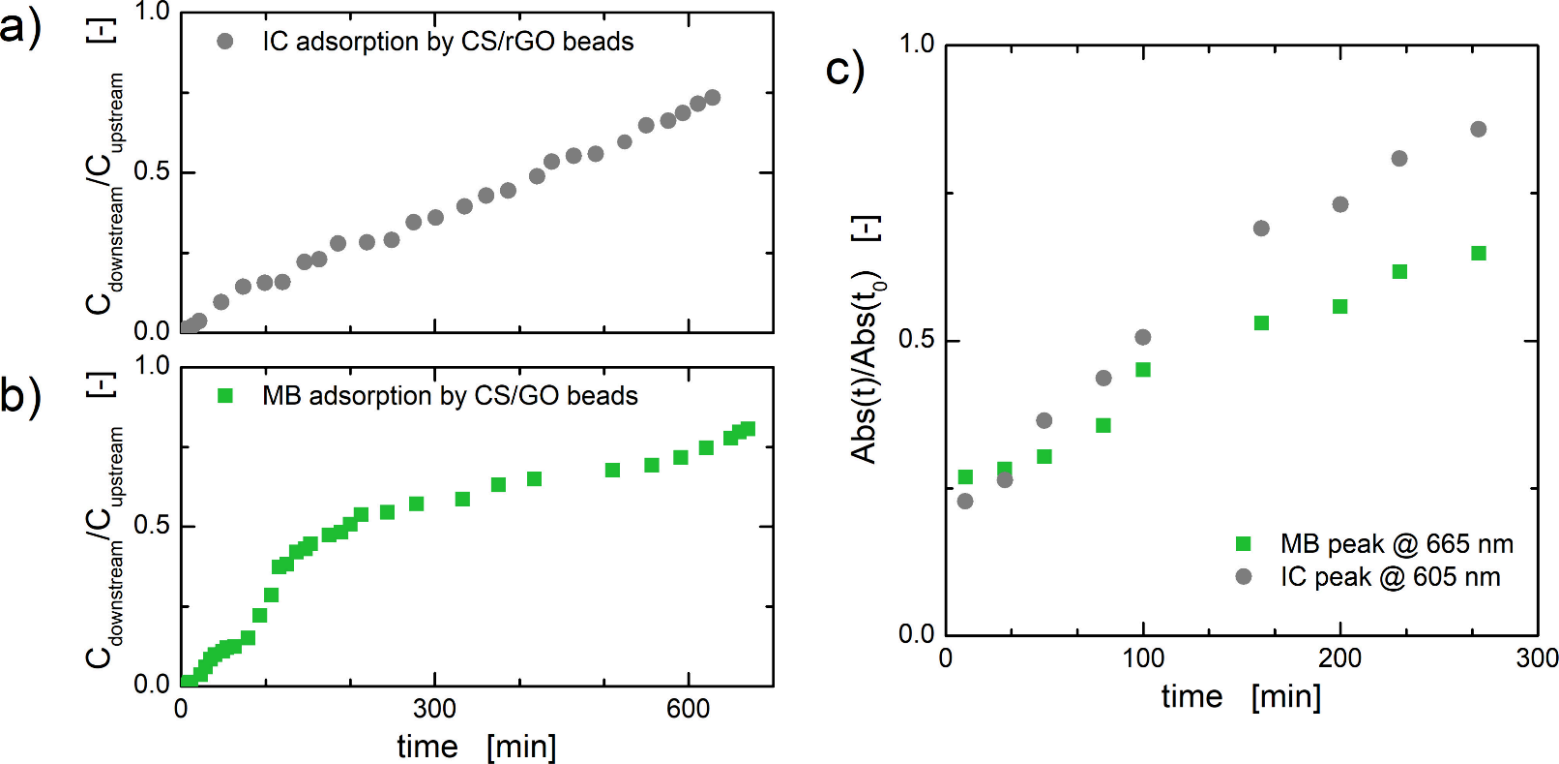
(a) Breakthrough curves for (a) IC using CS/rGO aerogel beads and (b) MB using CS/GO aerogel beads. (c) Normalized absorbance peak for MB and IC dyes in mixed solution during column adsorption using mixed CS/GO:CS/rGO beads (raw data are reported in Figure S3 of the Supporting Information).

For both the investigated materials, rapid adsorption of dye molecules was observed in the first half hour, and then the *C*_downstream_/*C*_upstream_ ratio began to increase, indicating a slow and steady decrease in the number of available adsorption sites. The maximum adsorption capacity, *q*_tot_, for a given column can be calculated as

$$q_{\mathrm{tot}}=\frac{1}{m}Q\int_{0}^{t_{\mathrm{end}}}(C_{\mathrm{upstream}}-C_{\mathrm{downstream})}dt$$ 12

where *m* is the amount of beads in the columns and *Q* is the flow rate.

According to eq 12, the experimental adsorption capacity of aerogel beads under flow conditions was found to be 13.32 mg g^–1^ for CS/rGO with IC and 23.76 mg g^–1^ for CS/GO with MB.

Finally, after evaluating the adsorption capabilities of CS/rGO and CS/GO beads independently for single-dye solutions, we extended our investigation by testing the adsorption behavior of mixed CS/GO:CS/rGO beads (1:1 weight ratio) as a packing material in the presence of mixed cationic/anionic (1:1 concentration ratio) dye solution. This approach in part simulates the usual scenario of industrial wastewater, which usually consists of a complex mixture of colorants and not of individual colorants. The peak absorbance values, Abs­(*t*), at wavelengths of 605 and 665 nm for IC and MB, respectively, were monitored over time, as shown in Figure 10c. In agreement with the adsorption capacity values of column tests calculated for single dye with single adsorbent, removal of MB is higher than IC. Nonetheless, the overall effectiveness of the beads for both dyes was demonstrated by the fact that the peak absorbance values did not reach the value of the starting stock solution, Abs­(*t*_0_), even after 250 min of test.

### 3.9 Comparison of Adsorption Capacity

The maximum adsorption capacities of different microspheres and beads for the removal of IC and MB from aqueous solutions are summarized in Table 1. Although certain materials have higher adsorption capacities compared to the aerogel beads proposed in the present work, their practical application could be often limited due to factors such as cost, complex synthesis procedures, and difficult recovery processes. On the other hand, millimeter-sized aerogel beads are a viable alternative as they have relatively high adsorption capacities while overcoming these practical limitations.

**Table 1:** Comparison of the Maximum Dye Adsorption Capacity of CS/GO and CS/rGO Aerogel Beads with Literature Data on Comparable Systems^a^

adsorbent	type	dye	*Q*_max_ **[mg g** ^**–1**^ **]**	equilibrium time [min]	pH	*T* [°C]	ref.
CS/GO	microsphere	MB	23.26	35	7	25	^57^
alginate-sepiolite	beads	MB	55.49	900	6.5	25	^58^
GO/calcium alginate	beads	MB	181.81	n.a.	5.4	25	^59^
alginate-halloysite nanotube	beads	MB	250	120	10	25	^60^
chitin/clay microsphere	microspheres	MB	152	540	7	25	^61^
CS/GA	beads	IC	303.03	120	6	30	^62^
gamma/alumina	beads	IC	126	n.a.	7	25	^63^
CS/activated carbon	beads	IC	208.33	20	3	30	^64^
Ce(III)/chitosan/β-cyclodextrin	beads	IC	30.57	50	3	25	^65^
GO/polyethylenimine/PVA hydrogel	beads	IC	68.44	120	9	25	^66^
CS/GO aerogel	beads	MB	254	350	6.8	25	this work
CS/rGO aerogel	beads	IC	109	350	6.7	25	this work

a Maximum dye adsorption capacity taken as Langmuir fitting parameter *Q*_max_.

## 4 Conclusions

Nanocomposite aerogel beads made of chitosan (CS) and either graphene oxide (GO) or reduced graphene oxide (rGO) were prepared, and their use for water purification purposes was investigated. Aerogel beads were obtained following a simple three-step process, which envisages bead formation by physical cross-linking, followed by chemical cross-linking, and eventually freeze-drying to get porous adsorbents. The size distribution, morphology, and mechanical and chemical stability of the produced aerogel beads were investigated. To evaluate their potential as adsorbents, the aerogel beads were tested in a batch adsorption setting against two model dyes: methylene blue (MB) and indigo carmine (IC). The mixed aerogel beads (CS/GO and CS/rGO) demonstrated preferential adsorption capabilities: CS/GO showed a high affinity for the cationic dye MB, while CS/rGO was more effective for the anionic dye IC. In view of these outcomes, we used the aerogel beads as packing materials in a 15 mL-scale fixed-bed adsorption system to assess their performance. The adsorption capabilities for MB and IC were found to be 23.762 and 13.315 mg/g, respectively, at the initial concentration of 3.5 mg/L. Additionally, a column made of mixed CS/GO and CS/rGO beads (1:1 weight ratio) was found to successfully adsorb mixed dye solution, highlighting the potential for broad-spectrum pollutant removal.

## Supplementary Material





## Notes

UV–vis spectra of dye solutions, wettability of aerogel beads, fitting of adsorption kinetics, fitting of adsorption isotherms, and raw data for mixed dye adsorption (PDF)
Fixed-bed adsorption test (MP4)
