# Supplementary material for: Nanocomposite Chitosan@graphene Oxide-Based Aerogel Beads for Anionic and Cationic Dye Removal: Synthesis, Characterizations, and Complexed Interfacial Interactions in Batch and Column Studies
Source: ACS Omega. 2025 Jul 8;10(28):31077–89. doi: 10.1021/acsomega.5c04669 (PMC12290692; doi:10.1021/acsomega.5c04669)
Supplement: Supplementary file 1 [file ao5c04669_si_001.pdf]

Supporting Information

Nanocomposite chitosan@graphene oxide-based aerogel beads for anionic and cationic dye removal: Synthesis, characterizations and complexed interfacial interactions in batch and column studies

Shehryar Ahmad [a], Enrica Luzzi [a], Konstantinos N. Maroulas [b], George Z. Kyzas [b,*], Martina Salzano de Luna [a,*]

[a] *Department of Chemical, Materials, and Production Engineering (INSTM Consortium − UdR Naples), University of Naples Federico II, P.le Tecchio 80, 80125 Naples, Italy; shehryar.ahmad@unina.it (S.A.), enrica.luzzi@unina.it (E.L.), martina.salzanodeluna@unina.it (M.S.L.)*
[b] *Hephaestus Laboratory, School of Chemistry, Faculty of Sciences, Democritus University of Thrace, GR-65404 Kavala, Greece; kmaroula@chem.duth.gr (K.N.M.), kyzas@chem.duth.gr (G.Z.K.)*

*Corresponding authors: G.Z. Kyzas (kyzas@chem.duth.gr), M. Salzano de Luna (martina.salzanodeluna@unina.it)

CONTENT

- Section S1 – UV-vis spectra of dye solutions
- Section S2 – Stability of aerogel beads
- Section S3 – Fitting of adsorption kinetics
- Section S4 – Fitting of adsorption isotherms
- Section S5 – Raw data for mixed dye adsorption

<u>Section S1 – UV-vis spectra of dye solutions</u>

Figure S1 shows representative UV-vis spectra of IC and MB solutions showing absorbance peaks at 610 and 662 nm, respectively.

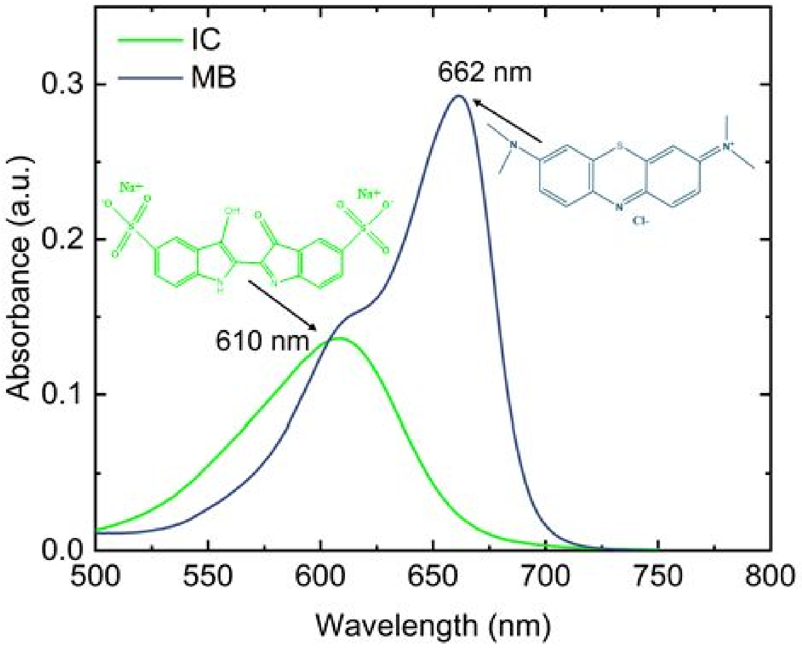

Figure S1. UV-vis spectra of the dye solutions, and corresponding chemical structures of the dye molecules.

Section S2 – Stability of aerogel beads

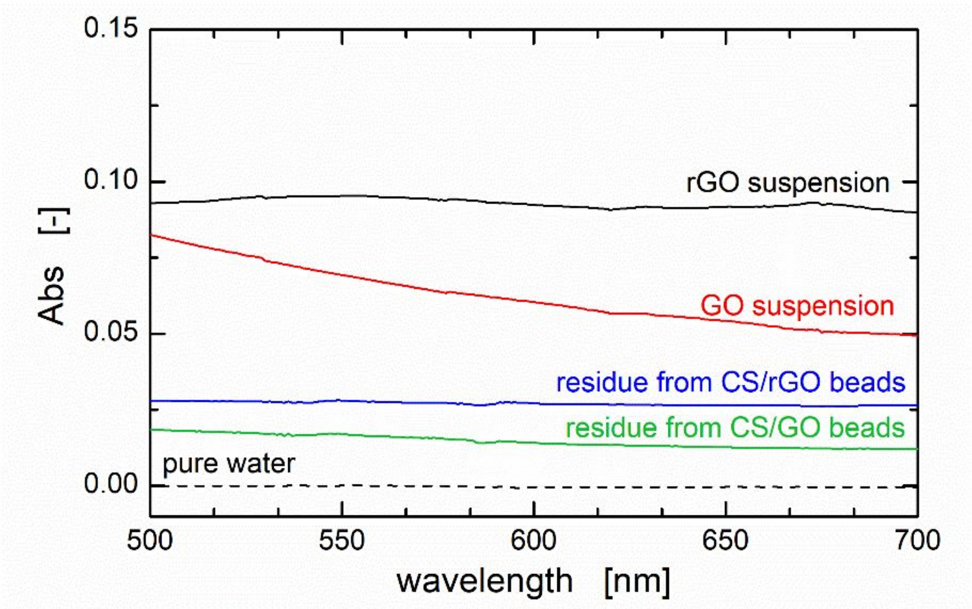

Figure S2. UV-vis spectra of water samples collected after soaking CS/GO and CS/rGO aerogel beads for 6 hours. Spectra of GO and rGO suspensions (~ 0.02 mg mL^{-1} and ~ 0.01 mg mL^{-1} respectively) are also included as reference.

Section S3 – Fitting of adsorption kinetics

Table S1. Best fitting parameters for the PFO kinetic model. Values in brackets are the standard error on parameters estimation.

Indigo Carmine				
**Adsorbent**	q_e **[mg g^{-1}]**	k_1 **[min^{-1}]**	**AdjR^2**	**SSE**
CS/GO	12.53 (0.25)	5.61E-02 (6.69E-03)	0.9731	3.885
CS/rGO	56.92 (3.50)	1.17 E-02 (2.13E-03)	0.8983	332.38
CS/GO:CS/rGO 1:3	49.18 (2.96)	8.74E-03 (1.46E-03)	0.9201	204.08
CS/GO:CS/rGO 1:1	43.56 (1.67)	7.26E-03 (7.49E-04)	0.9735	57.78
CS/GO:CS/rGO 3:1	26.21 (1.23)	9.08E-03 (1.19E-03)	0.9501	36.27

Methylene Blue				
Adsorbent	q_e [mg g^{-1}]	k_1 [min^{-1}]	AdjR^2	SSE
CS/GO	88.16 (6.92)	1.24E-02 (2.92E-03)	0.8258	1339.16
CS/rGO	56.45 (2.92)	2.11E-02 (3.88E-03)	0.8963	311.70
CS/GO:CS/rGO 1:3	56.16 (4.21)	2.02E-02 (5.27E-03)	0.7961	630.12
CS/GO:CS/rGO 1:1	64.54 (4.71)	1.52E-02 (3.52E-03)	0.8306	685.65
CS/GO:CS/rGO 3:1	79.25 (7.16)	7.88E-03 (1.93E-03)	0.8107	1115.37

R^2 = correlation coefficient
SSE = residual sum of squares error

Table S2. Best fitting parameters for the PSO kinetic model. Values in brackets are the standard error on parameters estimation.

Indigo Carmine				
Adsorbent	q_e [mg g^{-1}]	k_2 [g mg^{-1} min^{-1}]	AdjR^2	SSE
CS/GO	13.28 (0.31)	7.50E-03 (1.36E-03)	0.9760	3.4733
CS/rGO	63.79 (3.07)	2.42E-04 (5.01E-05)	0.9586	135.30
CS/GO:CS/rGO 1:3	56.67 (2.86)	1.83E-04 (3.67E-05)	0.9659	87.13
CS/GO:CS/rGO 1:1	51.57 (2.23)	1.52E-04 (2.48E-05)	0.9813	40.84
CS/GO:CS/rGO 3:1	30.20 (1.35)	3.53E-04 (6.26E-05)	0.9730	19.68

Methylene Blue				
Adsorbent	q_e [mg g^{-1}]	k_2 [g mg^{-1} min^{-1}]	AdjR^2	SSE
CS/GO	97.84 (6.97)	1.76E-04 (5.22E-05)	0.9134	666.37
CS/rGO	61.05 (2.67)	5.43E-04 (1.18E-04)	0.9526	142.72
CS/GO:CS/rGO 1:3	61.35 (4.13)	4.95E-04 (1.62E-04)	0.8945	326.01
CS/GO:CS/rGO 1:1	70.53 (4.55)	3.22E-04 (9.26E-05)	0.9171	335.82
CS/GO:CS/rGO 3:1	89.61 (8.80)	1.14E-04 (4.17E-05)	0.8774	722.64

R^2 = correlation coefficient
SSE = residual sum of squares error

Table S3. Best fitting parameters for the Elovich kinetic model. Values in brackets are the standard error on parameters estimation.

Indigo Carmine				
**Adsorbent**	**α [mg g^{-1} min]**	**β g mg^{-1}]**	**AdjR^2**	**SSE**
CS/GO	139.69 (294.57)	8.21E-01 (2.01E-01)	0.9008	14.32
CS/rGO	2.45 (0.24)	8.01E-02 (2.91E-03)	0.9952	15.65
CS/GO:CS/rGO 1:3	1.10 (0.12)	8.02E-02 (3.97E-03)	0.9920	20.32
CS/GO:CS/rGO 1:1	0.62 (0.11)	8.01E-02 (7.86E-03)	0.9740	56.69
CS/GO:CS/rGO 3:1	0.61 (0.11)	1.53E-01 (1.24E-02)	0.9782	15.81

Methylene Blue				
**Adsorbent**	**α [mg g^{-1} min]**	**β [g mg^{-1}]**	**AdjR^2**	**SSE**
CS/GO	4.78 (0.97)	5.66E-02 (3.85E-03)	0.9815	142.26
CS/rGO	11.27 (4.04)	1.11E-01 (9.35E-03)	0.97352	79.67
CS/GO:CS/rGO 1:3	9.12 (3.58)	1.02 E-01 (1.03E-02)	0.96384	111.79
CS/GO:CS/rGO 1:1	5.73 (1.52)	8.49E-03 (6.52E-03)	0.97646	95.35
CS/GO:CS/rGO 3:1	2.12 (0.64)	5.67E-03 (7.03E-03)	0.94336	333.81

R^2 = correlation coefficient
SSE = residual sum of squares error

Table S4. Best fitting parameters for the Intraparticle diffusion kinetic model. Values in brackets are the standard error on parameters estimation.

Indigo Carmine				
**Adsorbent**	**k_{dif} [mg g^{-1} min$^{-1/2}$]**	**c [mg g^{-1}]**	**AdjR^2**	**SSE**
CS/GO	0.27 (0.10)	7.15 (1.61)	0.3521	93.63
CS/rGO	1.90 (0.19)	12.35 (2.91)	0.9068	304.65
CS/GO:CS/rGO 1:3	1.72 (0.12)	6.96 (1.92)	0.9481	132.52
CS/GO:CS/rGO 1:1	1.56 (0.15)	3.61 (2.28)	0.9143	187.32
CS/GO:CS/rGO 3:1	0.90 (0.08)	3.99 (1.32)	0.9130	63.32

Methylene Blue				
Adsorbent	k_{dif} [mg g^{-1} min$^{-1/2}$]	c [mg g^{-1}]	AdjR^2	SSE
CS/GO	2.93 (0.28)	20.86 (4.35)	0.91136	681.60
CS/rGO	1.65 (0.32)	20.77 (4.86)	0.71784	848.84
CS/GO:CS/rGO 1:3	1.74 (0.28)	19.34 (4.31)	0.78409	667.47
CS/GO:CS/rGO 1:1	2.05 (0.27)	18.62 (4.22)	0.84152	641.80
CS/GO:CS/rGO 3:1	2.62 (0.17)	13.01 (2.65)	0.9569	253.98

R^2 = correlation coefficient
SSE = residual sum of squares error

Section S4 – Fitting of adsorption isotherms

Table S5. Best fitting parameters for the Langmuir isotherm model. Values in brackets are the standard error on parameters estimation.

Indigo Carmine				
Adsorbent	q_L [mg g^{-1}]	k_L [L mg^{-1}]	AdjR^2	SSE
CS/GO	211.9 (123.6)	4.50E-04 (3.28E-04)	0.9677	54.67
CS/rGO	109.4 (11.8)	1.87E-02 (8.68E-03)	0.8932	748.88
CS/GO:CS/rGO 1:3	131.8 (19.8)	5.29E-03 (2.14E-03)	0.9387	409.43
CS/GO:CS/rGO 1:1	137.4 (26.5)	3.26E-03 (1.40E-03)	0.9391	339.94
CS/GO:CS/rGO 3:1	141.7 (33.3)	2.21E-03 (9.95E-04)	0.9417	258.35

Methylene Blue				
Adsorbent	q_L [mg g^{-1}]	k_L [L mg^{-1}]	AdjR^2	SSE
CS/GO	254.8 (50.2)	7.27E-03 (3.87E-03)	0.8868	2909.34
CS/rGO	86.3 (1.3)	8.43E-02 (9.66E-03)	0.9944	28.48
CS/GO:CS/rGO 1:3	104.1 (5.3)	3.24E-02 (9.46E-03)	0.9629	263.49
CS/GO:CS/rGO 1:1	172.5 (17.4)	9.08E-03 (3.02E-03)	0.9544	664.11
CS/GO:CS/rGO 3:1	200.6 (28.2)	8.52E-03 (3.73E-03)	0.9201	1501.21

R^2 = correlation coefficient
SSE = residual sum of squares error

Table S6. Best fitting parameters for the Freundlich isotherm model. Values in brackets are the standard error on parameters estimation.

Indigo Carmine				
**Adsorbent**	k_F **[(mg g^{-1}) (mg L^{-1})$^{-1/n}$]**	n_F **[-]**	**AdjR^2**	**SSE**
CS/GO	0.28 (0.13)	1.26 (0.12)	0.9788	35.79
CS/rGO	15.84 (2.97)	3.27 (0.36)	0.9797	142.14
CS/GO:CS/rGO 1:3	6.52 (1.24)	2.31 (0.17)	0.9893	71.40
CS/GO:CS/rGO 1:1	3.77 (0.95)	2.00 (0.17)	0.9855	81.01
CS/GO:CS/rGO 3:1	2.27 (0.71)	1.79 (0.17)	0.9811	83.66

Methylene Blue				
**Adsorbent**	k_F **[(mg g^{-1}) (mg L^{-1})$^{-1/n}$]**	n_F **[-]**	**AdjR^2**	**SSE**
CS/GO	21.94 (7.49)	2.80 (0.49)	0.9513	1251.8
CS/rGO	34.79 (4.30)	6.75 (1.01)	0.9789	107.78
CS/GO:CS/rGO 1:3	24.78 (3.12)	4.39 (0.43)	0.9869	93.47
CS/GO:CS/rGO 1:1	14.38 (1.32)	2.69 (0.12)	0.9966	50.08
CS/GO:CS/rGO 3:1	16.47 (2.66)	2.69 (0.21)	0.9886	214.84

R^2 = correlation coefficient
SSE = residual sum of squares error

Table S7. Best fitting parameters for the Temkin isotherm model. Values in brackets are the standard error on parameters estimation

Indigo Carmine				
**Adsorbent**	k_T **[L g^{-1}]**	RT/b_t **[J mol^{-1}]**	**AdjR^2**	**SSE**
CS/GO	0.01	17.38 (3.13)	0.8903	185.30
CS/rGO	0.32 (0.21)	19.84 (3.16)	0.9479	365.08
CS/GO:CS/rGO 1:3	0.09 (0.04)	23.70 (3.49)	0.9432	379.18

CS/GO:CS/rGO 1:1	0.06 (0.02)	23.64 (3.81)	0.9280	401.50
CS/GO:CS/rGO 3:1	0.04 (0.01)	23.15 (3.97)	0.9160	371.79

**Methylene Blue**				
**Adsorbent**	k_T **[L g^{-1}]**	RT/b_t **[J mol^{-1}]**	**AdjR^2**	**SSE**
CS/GO	4.60 (7.79)	21.61 (5.95)	0.8112	4848.56
CS/rGO	7.37 (4.62)	10.63 (0.93)	0.9901	51.04
CS/GO:CS/rGO 1:3	0.80 (0.28)	16.80 (1.16)	0.9905	0.6692
CS/GO:CS/rGO 1:1	0.18 (0.06)	30.66 (3.29)	0.9673	475.76
CS/GO:CS/rGO 3:1	0.28 (0.17)	31.19 (4.91)	0.9292	1329.31

R^2 = correlation coefficient
SSE = residual sum of squares error

Table S8. Best fitting parameters for the Sips isotherm model. Values in brackets are the standard error on parameters estimation.

**Indigo Carmine**					
**Adsorbent**	q_s **[mg g^{-1}]**	k_s **[L mg^{-1}]**	n_s **[-]**	**AdjR^2**	**SSE**
CS/GO	212 (1999.1)	2.25E-9 (3.18E-6)	5.00E-6 (7.15E-3)	0.9595	54.66
CS/rGO	109.4 (25.51)	2.15E-5 (5.94E-5)	1.1E-3 (3.11E-1)	0.8665	748.8
CS/GO:CS/rGO 1:3	131.8 (55.69)	2.00E-7 (4.55E-5)	3.79E-5 (8.64E-3)	0.9233	409.4
CS/GO:CS/rGO 1:1	137.4 (90.15)	2.42E-5 (0.00647)	7.41E-3 (1.99E-1)	0.9238	339.9
CS/GO:CS/rGO 3:1	141.8 (142.4)	2.78E-8 (8.97E-6)	1.26E-5 (4.1E-3)	0.9271	258.3

**Methylene Blue**					
**Adsorbent**	q_s **[mg g^{-1}]**	k_s **[L mg^{-1}]**	n_s **[-]**	**AdjR^2**	**SSE**
CS/GO	254.8 (147.5)	2.96E-13 (1.0E-10)	4.08E-11(1.39E-8)	0.8584	2909.3
CS/rGO	86.29 (2.12)	2.79E-5 (2.38E-3)	3.31E-4 (2.82E-3)	0.9930	28.48
CS/GO:CS/rGO 1:3	104.0 (9.20)	2.71E-4 (4.05E-2)	8.38E-3 (1.25)	0.9536	263.4
CS/GO:CS/rGO 1:1	172.4 (41.43)	1.87E-4 (3.416E-2)	2.063E-3 (3.77)	0.9429	664.1
CS/GO:CS/rGO 3:1	200.6 (72.37)	3.28E-5 (8.46E-2)	3.85E-3 (0.99)	0.9001	1501.2

R^2 = correlation coefficient
SSE = residual sum of squares error

Section S5 – Raw data for mixed dye adsorption

Figure S3 shows the UV-vis spectra over time of mixed dye solution collected after passing through the column packed with mixed CS/GO and CS/rGO beads.

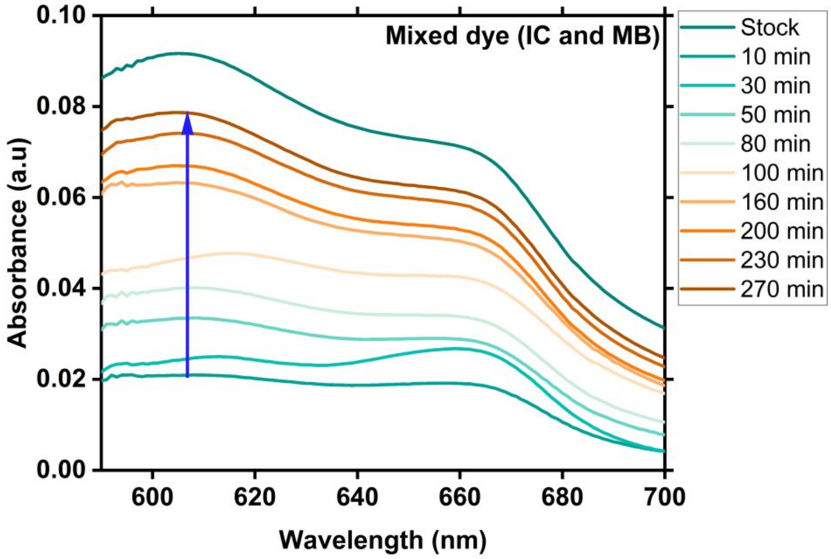

Figure S3. UV-vis spectra of samples collected after the column containing mixed CS/GO and CS/rGO beads.
